# New horizons in smart plant sensors: key technologies, applications, and prospects

**DOI:** 10.3389/fpls.2024.1490801

**Published:** 2025-01-07

**Authors:** Fucheng Zhang, Denghua Li, Ganqiong Li, Shiwei Xu

**Affiliations:** ^1^ Research Center for Agricultural Monitoring and Early Warning, Agricultural Information Institute of Chinese Academy of Agricultural Sciences, Beijing, China; ^2^ Key Laboratory of Agricultural Monitoring and Early Warning Technology, Ministry of Agriculture and Rural Affairs, Beijing, China; ^3^ Research Center of Agricultural Monitoring and Early Warning Engineering Technology, Beijing, China

**Keywords:** smart planting, sensors, nanotechnology, wearable plant sensors, intelligent monitoring, multimodal sensors

## Abstract

As the source of data acquisition, sensors provide basic data support for crop planting decision management and play a foundational role in developing smart planting. Accurate, stable, and deployable on-site sensors make intelligent monitoring of various planting scenarios possible. Recent breakthroughs in plant advanced sensors and the rapid development of intelligent manufacturing and artificial intelligence (AI) have driven sensors towards miniaturization, intelligence, and multi-modality. This review outlines the key technologies in developing new advanced sensors, such as micro-nano technology, flexible electronics technology, and micro-electromechanical system technology. The latest technological frontiers and development trends in sensor principles, fabrication processes, and performance parameters in soil and different segmented crop scenarios are systematically expounded. Finally, future opportunities, challenges, and prospects are discussed. We anticipate that introducing advanced technologies like nanotechnology and AI will rapidly and radically revolutionize the accuracy and intelligence of agricultural sensors, leading to new levels of innovation.

## 1 Introduction

With the widespread application of modern information technology in the agricultural sector, crop cultivation methods are gradually transitioning from traditional to smart farming (Karunathilake et al., 2023). This transition offers favorable conditions to meet the demands of food security and sustainable agricultural development in the new era (Yang X. et al., 2021; Jararweh et al., 2023). Sensors act as the “senses” of smart agriculture and serve as the medium for information acquisition (Paul et al., 2022; Soussi et al., 2024). In the realm of smart farming, crop sensors, and soil sensors form the critical foundation for data acquisition and intelligent decision-making management. They play a pivotal role in the real-time monitoring of crop growth conditions on internal factors, such as biochemical information in tissues or cells (Bakhori et al., 2013), health characteristics (Bayrakdar, 2019), and growth rates (Kim et al., 2019), as well as external environmental factors that affect plant growth, including soil moisture (Aiello et al., 2018; Boada et al., 2018) and nutrient status (Guerrero et al., 2021). For instance, Grell et al. (2021) developed and used novel, low-cost point-of-use (PoU) NH_4_^+^ sensors for soil fertilization management. Each sensor costing less than $0.10. It enables real-time detection of NH_4_^+^ content in soil, with a detection limit of 3 ± 1 ppm. The researchers demonstrated that point-of-use measurements of NH_4_^+^, combined with soil conductivity, pH, weather and timing data, allow instantaneous prediction of levels of NO_3_^−^ in soil. By using this type of sensor, famers can forecast the impact of climate on fertilization planning and to tune timing for crop requirements, reducing overfertilization while improving crop yields. Lew et al. (2020) developed a nanosensor based on single-walled carbon nanotubes (SWNTs) for real-time detection of hydrogen peroxide (H_2_O_2_) induced by plant wounds. This sensor demonstrates high sensitivity (≈ 8 nm ppm^-1^) and utility and can be interfaced with portable, cost-effective electronic devices, enabling real-time monitoring of plant health in the field. Based on multimodal crop and soil information gathered by sensors in smart farming, farmers can monitor, analyze, and even predict crop growth and yield in real-time. This enables targeted and precise input of agricultural resources, such as water, fertilizers, and pesticides, leading to refined crop management, intelligent operations, and scientific decision-making. Ultimately, this approach enhances cultivation efficiency and promotes the sustainable development of agriculture (Idoje et al., 2021).

In recent years, driven by innovations in micro-nano sensing technology (Liu et al., 2020), flexible electronics (Rim et al., 2016), biotechnology (He et al., 2014), and other fields, the development of smart planting sensors has ushered in new opportunities. More and more new theories, technologies, and materials have been developed and applied in the agricultural sensor field, with new advanced sensors continuously emerging (Bock et al., 2020; Shaw and Honeychurch, 2022). The development of new advanced sensors for crop planting involves multiple disciplines such as crop physiology, electronics, materials science, and computer science, with distinct multidisciplinary integration characteristics (Ojha et al., 2015; Cao X. et al., 2023). For example, advanced technologies such as micro-nano technology and flexible electronics empower agricultural sensors, promoting the development of a batch of wearable crop life information sensors. These sensors have flexible adhesion and can be installed on the irregular surfaces of crop tissues for *in-situ*, real-time, continuous precise monitoring (Mao et al., 2023); the development of wireless network sensing technology provides strong support for remote monitoring and early warning and control of agricultural environments (such as nutrients, temperature, humidity, moisture content, pH value, heavy metals, etc.) (Yin et al., 2021); the combination of artificial intelligence, machine learning, and hyperspectral sensing technology offers new ideas for crop disease monitoring (Terentev et al., 2022), growth monitoring (Lin et al., 2019), yield estimation (Jin et al., 2020), and quick detection of agricultural product quality (Li Y. et al., 2022).

Currently, some excellent review articles on advanced agricultural sensors focus on specific aspects of sensing methods and technologies, such as wearable technology (Lee et al., 2021; Qu et al., 2021b), nanotechnology (Lombi et al., 2019), and non-invasive technology (Ang and Lew, 2022; Presti et al., 2023). However, there is still a lack of comprehensive reviews specifically targeting various new advanced sensors in the segmented field of crop planting. Moreover, with the rapid daily advancements in new principles, processes, and methods of new advanced sensors for smart planting, new collection methods, and new sensors for specific information in various segmented scenarios of smart planting have made new progress. Hence, it is necessary to overview and comparatively analyze the progress of new advanced sensors for smart planting in recent years to clarify the challenges that need to be overcome in this field.

**Figure 1:**
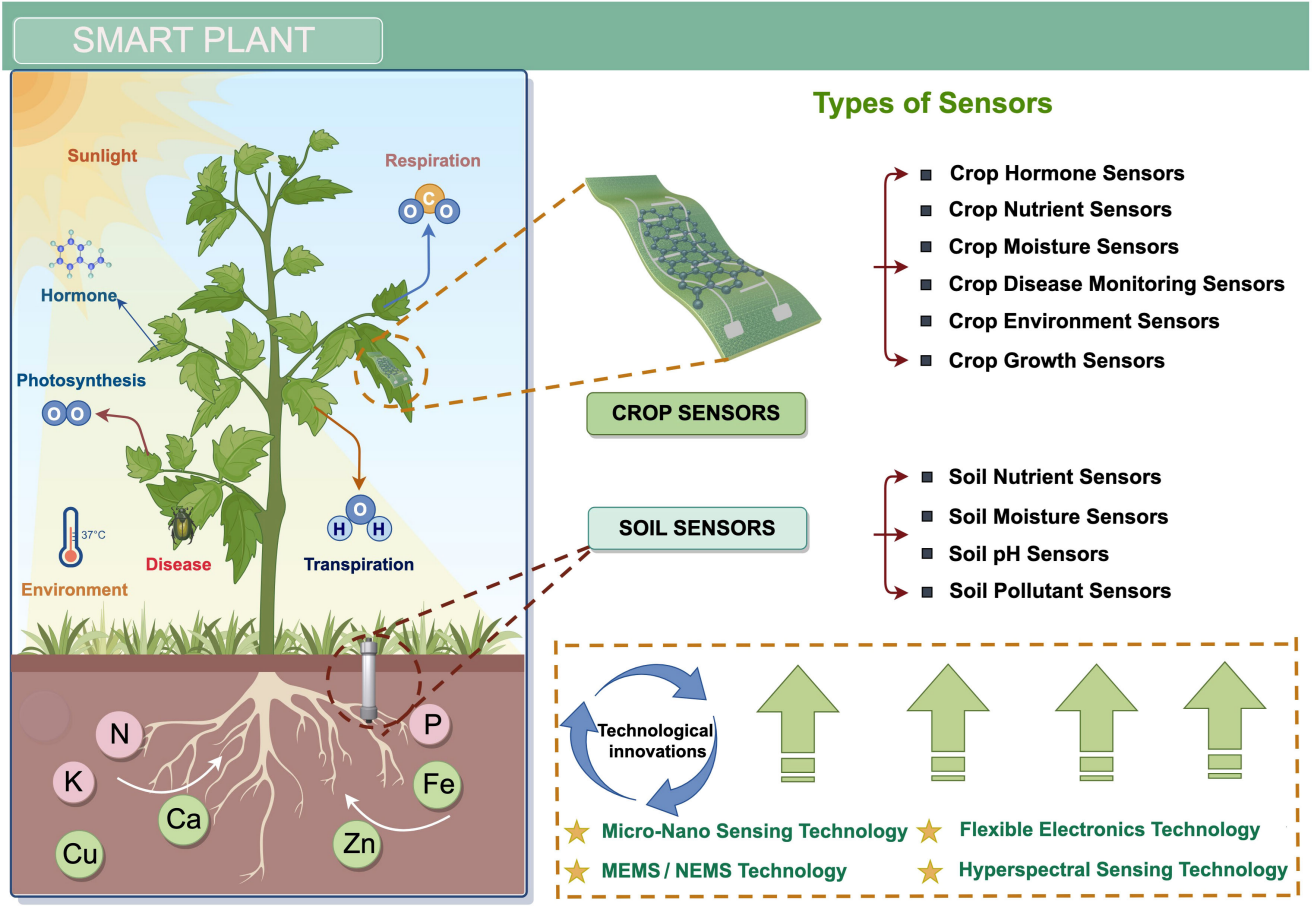
Architecture diagram of an advanced sensor system for smart planting (By Figdraw).

This review focuses on the application scenarios of crops and soil involved in smart planting, summarizing the latest frontier advances in new advanced sensors for crop planting. It first introduces the key technologies to be applied in manufacturing advanced sensors such as micro-nano technology, flexible electronics, and MEMS technology. Next, it focuses on the latest research progress and technological frontiers of sensors in the field of crop planting in recent years. More specifically, it details new advanced sensors aimed at detecting nutrients (N, P, K), hormones (e.g., salicylic acid, ethylene) moisture, diseases, and crop growth deformation, as well as new advanced multimodal sensors designed to detect crop habitats (e.g., microclimate and soil). As shown in the crop planting key factor monitoring system framework in **Figure 1**, these monitoring objects are the key factors determining crop productivity and agricultural product quality during the crop planting process. We also conduct a comparative analysis of the sensing principles, fabrication processes, performance parameters, and representative applications of various latest sensors.

The reference literature for this review was primarily compiled utilizing the Web of Science database. The search was focused on significant advancements in crop sensor technology over recent years, with particular attention given to developments in the past five years, and especially the last three years. Additionally, seminal works from over five years ago that continue to influence current research were included. Specific keywords related to advanced crop sensors were employed to ensure a comprehensive search. The selection criteria were mainly based on the novelty and significance of the advancements presented in the papers. The collected literature was further filtered by examining the scientific theories and the relevance of the research to our review objectives. The papers were then scrutinized to extract critical elements such as objectives, methods, results, and any remaining challenges or unraised issues. A thorough synthesis of the findings and comparisons of parameters were performed to present a cohesive overview of the current landscape in crop sensor technology. By applying these criteria, it was aimed to ensure that our review reflects a balanced and comprehensive perspective on recent advancements in the field.

In this review, section 2 discusses the key technologies used in manufacturing advanced sensors. Section 3 delves into the latest advancements in sensor applications for crop planting. Section 4 presents a comparative analysis of multimodal sensors. Finally, in section 5, the challenges faced by crop sensors are analyzed and discussed, and the future prospects are envisioned, such as multimodal sensing, intelligent data analysis within sensors, and artificial intelligence sensors, providing valuable insights for guiding the development of new advanced sensors for crops in the future. We believe this comprehensive review will offer a new perspective to observe and design agricultural sensors to enhance productivity.

## 2 Advanced sensing technology

The application environments (water, air, and soil) and monitoring objects (plants and animals) of agricultural sensors are diverse and complex, characterized by high temporal variability. Therefore, agricultural sensors with high environmental adaptability, high reliability, high precision, and low cost are key to realizing intelligent perception of crop information (Shaikh et al., 2022; Musa et al., 2024). Advancements in sensor technology have facilitated the monitoring of crop conditions. Currently, with the development of various emerging technologies such as micro-nano sensing technology, flexible electronics technology, micro-electro-mechanical systems (MEMS) technology, agricultural sensors have driven significant progress in high-precision monitoring, flexible wearable monitoring, and multi-parameter integrated monitoring. These emerging technologies play a pivotal role in facilitating the construction of advanced agricultural sensors.

### 2.1 Micro-nano sensing technology

**Figure 2:**
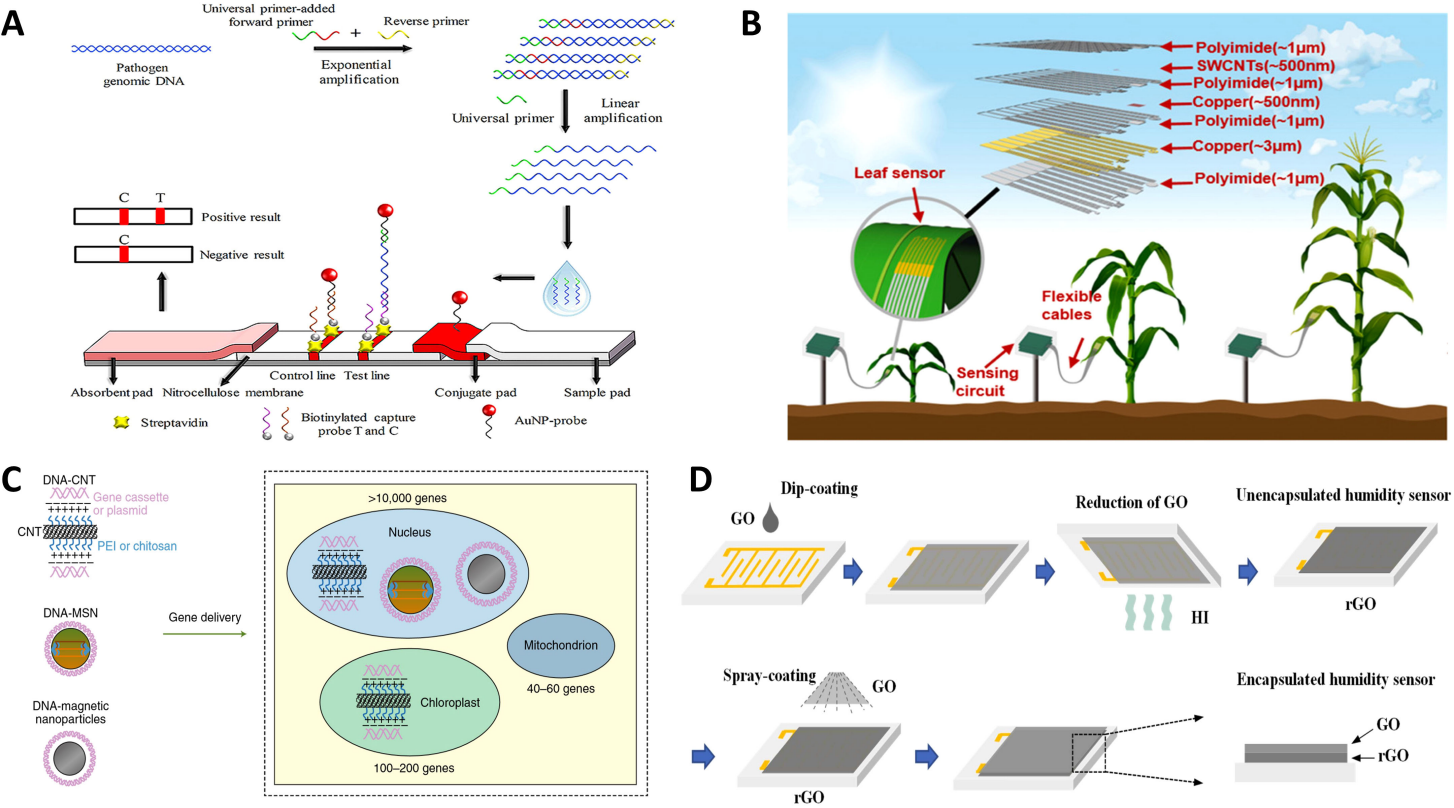
Example diagram of sensors developed based on micro nano sensing technology. **(A)** Schematic illustration of gold nanoparticle-based lateral flow biosensor for identification of plant pathogens (Zhan et al., 2018). **(B)** Schematic diagram of manufacturing and application demonstration of a multifunctional stretchable sensor on a leaf (Zhao et al., 2019). **(C)** Nanomaterials coated in DNA can be utilized as delivery systems of gene cassettes and plasmids to nuclear, chloroplast and mitochondrion genomes (Giraldo et al., 2019). **(D)** Schematic diagram of the preparation of the encapsulated humidity sensor by using film coating of interfinger electrodes (Huang et al., 2023).

Micro-nano sensing technology integrates nanomaterials and nanoprocesses with traditional sensing technologies to achieve high-precision recognition and monitoring of small signals, making it one of the key technologies in advanced sensor manufacturing (Luo et al., 2018; Lew et al., 2020). Traditional sensing technologies have proven effective in perceiving plant phenotypic information at macroscopic scales, such as canopy information, chlorophyll and nitrogen content, leaf area index, and incidence of diseases and pests. However, capturing critical information about plant responses to environmental stresses and changes in internal physiological signals at the micro-nano scale remains a challenge (Zhang Q. et al., 2022). Therefore, incorporating micro-nano technology into the design of sensors is expected to enhance the detection range, sensitivity, selectivity, and response speed of agricultural sensors, thereby aiding in the intuitive understanding of plants’ physiological states and their dynamic responses to environmental changes (Giraldo et al., 2019). In recent years, micro-nano manufacturing technology has continued to advance, driving more sophisticated sensor designs and performance improvements. As shown in **Figure 2**, the fabrication process of micro-nano sensors includes modification and assembly of nano particle probes (**Figure 2A**), printable electronics and transfer printing techniques (**Figure 2B**), nanomaterials-DNA composite assembly (**Figure 2C**), and film coating of interfinger electrodes (**Figure 2D**). Each type of sensor exhibits various advantages in terms of sensing mechanism, specificity, resolution, response speed, and manufacturing cost.

Nanotechnology offers unique advantages in designing and preparing intelligent plant sensors. First, nanomaterials exhibit surface size effects, providing high sensitivity and precision when used as sensitive materials (Wang et al., 2004; Qian et al., 2015). Biosensors based on micro-nano sensing technology offer higher measurement precision and sensitivity (Nissler et al., 2022), with detection limits reaching ppm and even ppb levels (Lupan et al., 2016). These sensors can achieve high temporal and spatial resolution for monitoring agricultural production environments and physiological parameters of plants and animal (Zhang et al., 2021). Under safe and controlled conditions, nanomaterials can be embedded into plants to monitor internal signaling molecules in real-time through the fluorescence signal. Nanomaterials can also serve as DNA scaffolds, overcoming plant cell barriers to provide gene-encoded biosensors for crop research (Yang et al., 2014; Dai et al., 2019). Nanotechnology also facilitates the design of plant wearable sensors. For instance, the integration of graphene-based sensor arrays with flexible silver nanowire electrodes onto a stretchable substrate facilitates the fabrication of a chemical resistive sensor array that can adhere to leaf surfaces. This array enables real-time identification of volatile organic compounds (VOCs), allowing for non-invasive early diagnosis of plant diseases (Li Z. et al., 2021). The aforementioned nanotechnology-based methods offer a powerful sensing tool to help us achieve precise, intelligent, and sustainable agriculture.

### 2.2 Flexible electronics technology

**Figure 3:**
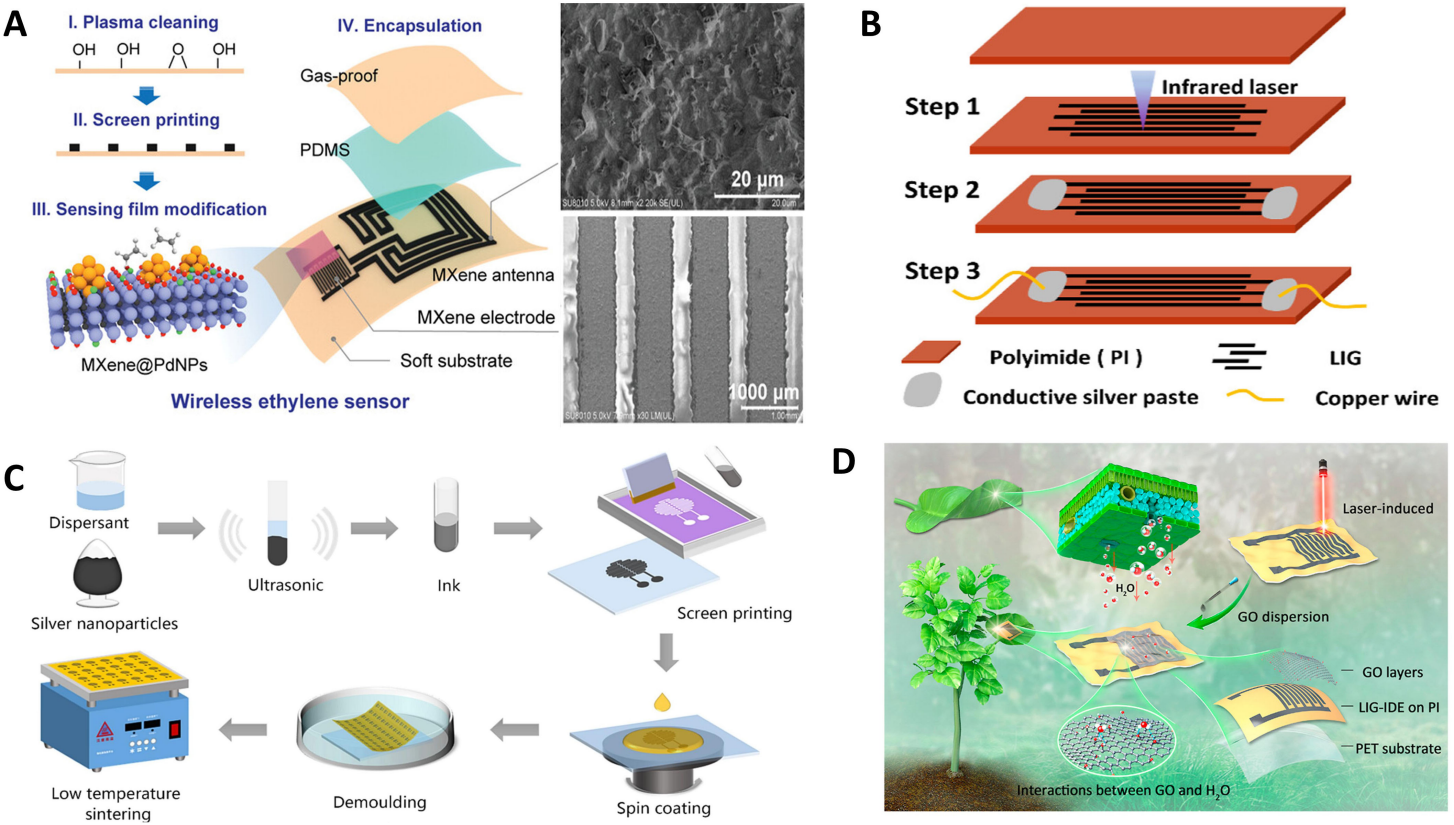
Example diagram of flexible sensor. **(A)** Schematic diagram of the fabrication process of sensors based on All-MXene Printed RF resonators using screen printing technology (Li et al., 2023). **(B)** Schematic of the fabrication process of the laser-induced graphene (LIG) humidity sensor (Liu et al., 2023). **(C)** Overview of the preparation of semi-embedded flexible multifunctional sensor for monitoring plant microclimate (Qu et al., 2024a). **(D)** The figure shows a one-step large-scale manufacturing method for a flexible wearable humidity sensor based on laser-induced graphene (Lan et al., 2020).

Flexible electronics technology is an emerging electronics technology that integrates electronic devices onto flexible/stretchable substrates. Its advent addresses the poor compatibility issue between traditional rigid sensors and measurement points (Qu et al., 2021b). Flexible electronic devices provide a new approach to the development of *in-situ* monitoring sensors for crop information. Advanced agricultural sensors developed using flexible electronics technology exhibit excellent stretchability and high biocompatibility (**Figure 3A**). They conform well to the surface of crops and adapt to the stretching and deformation of tissues and organs (Zhao et al., 2020). Currently, plant flexible sensors can monitor various types of information in growing crops, such as electrical signals (Luo et al., 2021), volatile chemicals (Li Z. et al., 2021), health status (Li et al., 2019), moisture content (Oren et al., 2017), growth rate (Tang et al., 2019), as well as microclimate conditions like surface temperature, humidity, and illumination (Nassar et al., 2018).

Flexible sensors are typically fabricated using techniques such as lithography, printing, roll-to-roll manufacturing processes, and laser direct writing (Gong et al., 2022). Photolithography represents one of the key technologies in the fabrication of flexible electronic devices, owing to its advantage in precisely defining and preparing microelectronic devices at a small scale (Cui et al., 2024). Bathaei et al. (2023) employed photolithographic processes to pattern metal layers onto flexible and stretchable substrates, including polylactic acid (PLA) and polyglycerol sebacate (PGS). Within a footprint of 1 square centimeter, they achieved the fabrication of up to 1600 devices, which can be integrated into various biodegradable passive electrical components, mechanical sensors, and chemical sensors. In the fabrication of micro/nano patterns, common printing techniques encompass soft lithography, nanoimprint lithography, screen printing, inkjet printing, and direct ink writing (DIW) 3D printing. Each technique boasts its unique merits and application scenarios (Yan et al., 2024). For instance, soft lithography achieves pattern transfer through the integration of stamps and self-assembled monolayers (Qin et al., 2010). By designing various stamps or templates, the fabrication of intricate patterns can be readily accomplished, rendering this technique suitable for patterning large areas and complex surfaces. Laser direct writing technology employs a laser beam to perform variable-dose exposure of the photoresist material on the substrate surface, resulting in the formation of the desired relief profile on the resist layer after development. This method offers advantages such as straightforward fabrication process and low cost, with manufacturing precision achievable at the sub-micrometer level (**Figure 3B**). Screen printing, a traditional printing method, employs various inks to directly print onto curved substrates (**Figure 3C**), characterized by its simplicity in principle and low cost (Meder et al., 2021). DIW 3D printing, an extrusion-based additive manufacturing approach, exhibits a high degree of complementarity with the production of soft, stretchable, and shape-retaining biophysical sensors for wearable applications, offering a high level of freedom for rapid prototyping of micro/nanostructures (Tay et al., 2023). In recent years, the laser direct writing technology has made remarkable progress and achieved widespread applications in the field of agricultural flexible electronics, driven by the increasing demand for miniaturization and flexibility of electronic devices (Coelho et al., 2022). Lan et al. (2020) fabricated a humidity sensor utilizing laser-induced graphene (LIG) interdigital electrodes (LIG-IDEs) on polyimide (PI) films (**Figure 3D**). The porous LIG served as the flexible electrode, while graphene oxide (GO) acted as the humidity-sensing material. This one-step fabrication process significantly facilitates large-scale production for practical applications. Li M. et al. (2021) using a direct laser writing instrument, simple and disposable LIPG (Laser-Induced Porous Graphene) electrodes were fabricated on PI (Polyimide) films, exhibiting satisfactory stability and flexibility. Subsequently, these electrodes were integrated with a portable micro-potentiostat equipped with Bluetooth wireless communication capabilities, a tablet computer, to develop a wireless smart flexible micro-sensor. The sensor demonstrated a wide linear range extending from 0.5 to 500 μM, a low limit of detection (LOD) of 0.16 μM, and a sensitivity of 10.99 μA μM^-1^.

The materials used for fabrication mainly fall into three categories: substrate materials, sensing materials, and encapsulation materials (Nan et al., 2022). Substrate materials support the functional circuits of flexible sensors and generally have good stretchability and bendability. Commonly used substrate materials include polydimethylsiloxane (PDMS), Eco-flex, polyimide (PI), and biodegradable materials. Sensing materials are the core of flexible sensors and determine their performance and characteristics. They are generally composed of conductive or semiconductor materials and are processed into functional films using methods such as spin coating (Oren et al., 2017), sputter deposition (Zhao et al., 2019), and chemical vapor deposition (Kim et al., 2019). Encapsulation of flexible wearable sensors is crucial (Li et al., 2020; Nguyen et al., 2021). The selection of encapsulation materials is dependent upon the specific application scenario. The materials must possess the requisite properties, such as waterproof, light-transmissive, breathable, or electromagnetic shielding properties, to ensure the realization of sensor functions and maintain performance stability in complex environments.

### 2.3 Micro-electro-mechanical systems/nano-electro-mechanical systems

Micro/Nano Electro-Mechanical Systems (MEMS/NEMS) represent the integration of electronic and mechanical components at the micrometer and nanometer scales (Sun et al., 2018). As an advanced microfabrication technology, MEMS/NEMS can achieve processing accuracy at the micrometer or even nanometer scale, thereby enabling the batch and integrated production of nanoscale sensor devices. Compared to traditional manufacturing processes, agricultural sensors manufactured using MEMS/NEMS technology demonstrate higher sensitivity, shorter response times, smaller size, and lower power consumption. These advantages give them significant potential in high-throughput diagnostics of crop life information in agriculture. For example, MEMS/NEMS-based acoustic sensors can locate insect flight paths in two dimensions, enabling effective pesticide application and crop protection. Various MEMS devices have already permeated various agricultural fields, providing new solutions for soil property monitoring (Thomas et al., 2021), crop physiological index monitoring (Miner et al., 2017), livestock disease diagnosis (Fu et al., 2020), and intelligent agricultural machinery control (Si et al., 2019), driving agricultural sensors towards array-based, integrated, and intelligent development (Li D. et al., 2021). Notably, although MEMS/NEMS have been employed in several applications within the agricultural sector, their utilization in agriculture, especially in crop cultivation, remains relatively limited compared to other industries. However, due to the demand for improving agricultural processes and the widespread use of the Internet of Things (IoT) in the future, it is anticipated that there will be a high demand for small-sized, low-cost, low-power consumption, and easily mass-produced devices (Singh and Singh, 2020). Hence, ample opportunities exist for further advancements. Furthermore, the transition from rigid substrates in MEMS/NEMS to flexible substrates, which is intended to enhance the flexibility of deployment and compatibility of interfaces on crop plants, represents a significant trend in the development of the next generation of multifunctional crop sensors.

The advancement of sensing technology has given birth to a large number of advanced sensors of various categories. Through the integration and innovation of these technologies, agricultural sensors have not only witnessed improvements in performance but also experienced significant expansion in their application scope and depth. For instance, the integration of micro-nano sensing technology with flexible electronics has led to the development of high-performance wearable sensors. The successful amalgamation of wireless sensor networks with MEMS technology and self-powered technology enables smaller, lower-power sensor nodes to communicate with each other, facilitating their use in farmlands with extensive cultivation areas and difficult-to-access wiring. Furthermore, modern information technologies such as artificial intelligence, coupled with cloud platforms, offer efficient management and analytical tools for handling complex and vast agricultural-related information. This interdisciplinary technological convergence provides new directions and opportunities for sensor development, propelling the progress of smart sensors in various aspects, including crop information monitoring, environmental monitoring, and multi-modal information monitoring.

## 3 Smart sensors for monitoring crop information

The growth of crops is a highly dynamic process that is sensitive to changes in external conditions such as the growing environment and pest infestations. It also involves internal signals of hormone, moisture, and nutrient fluctuations, forming a complex interplay among these factors (Schans and Arntzen, 1991). To mitigate crop yield losses caused by various biotic and abiotic stresses, it is essential to select appropriate measurement tools that can promptly and dynamically acquire crop growth information, diagnose crop health status, and provide decision support for timely regulation (Lee et al., 2023). Technologies such as nano-sensors, flexible electronics, microneedle technology, and spectroscopy are currently being effectively employed in the development of crop sensors, leading to substantial advancements in sensor accuracy and stability. These innovations provide researchers with robust tools for non-invasive monitoring of crop health, precise tracking and prediction of crop diseases, and revealing physiological and biochemical processes in crops. **Supplementary Figure S1** shows bibliometric analysis of different kinds of crop sensors published over the past decade using the Web of Science database. **Table 1** summarizes the sensitive materials used in several types of crop sensors and compares key parameters like measurement accuracy and monitoring range.

**Table 1:** Crop sensors.

Application	Sensitive Materials	Detection Objects	Measurement Accuracy	Working Range	Response Time	Reference
Crop Hormone Monitoring	Au@Cu_2_O-Gr-PDA	Indole acetic acid	LOD: 0.00116μM	0.01 ~ 100μM	50 s	(Wu et al., 2022)
	MXene@PdNPs	Ethylene	LOD: 0.084 ppm	0.5 ~ 20 ppm	5 min	(Li et al., 2023)
	SWNT	Gibberellic acid	GA_3_: 542 nM GA_4_: 2.96 μM	0 ~ 150 μM	/	(Boonyaves et al., 2023)
	Magnetic molecularly imprinted polymers	Salicylic acid	LOD: 2.74 μM	0 ~ 20 and 50 ~ 150 μM	1.5 s	(Bukhamsin et al., 2022)
	Au@SnO_2_-vertical graphene	Abscisic acid	LOD:0.002 ~ 0.005 μM	0.012 ~ 495.2 μM	/	(Wang et al., 2021)
Crop Moisture Sensors	Graphene oxide	Corn leaf	Sensitivity: 7945 Ω/%RH	11 ~ 95% RH	20.3 s	(Li D. et al., 2022)
	Positive temperature coefficient (PTC) thermistor	Watermelon stemflow	Sensitivity: 5 μL min^-1^	5 ~ 415 μL min^-1^	30 s	(Chai et al., 2021)
	Ni films and ecofriendly pyrolyzed paper	Soy plants	Sensitivity: 27.0 kΩ %^-1^	0~70% and 70~90% LWC	2 s	(Barbosa et al., 2022)
	Laser-induced graphene (LIG)	Corn leaf	Sensitivity: 0.042, 0.104Ω%^-1^	25~100% RH	/	(Yin et al., 2021)
Crop Environment Sensors	Humidity: Nafion Temp: Au@AgNWs	Humidity, temperature	/	Temp: 20~60°C Humidity: 0~100% RH	/	(Lee et al., 2023)
	Humidity: Fmwcnt/HEC/PVPP Temperature: PEDOT: PSS/GOPS	Humidity, temperature	Temp: 10.5478°C Humidity: 11.321% RH	Temp: 10 ~ 90°C Humidity: 10 ~ 90% RH	<1 min	(Hossain and Tabassum, 2023)
Crop Disease Monitoring	AuNP@rGO	late blight	LOD: 0.17 to 3.9 ppm	10 ~ 50 ppm	/	(Li Z. et al., 2021)
	DμFED/Ab_1_/CP/Ab_2_/MB/HRP	Citrus tristeza virus (CTV)	LOD: 0.3 fg mL^-1^	1.95 ~ 10.0 × 10^3 fg mL^-1^	/	(Freitas et al., 2019)
	GNP	Phytophthora infestans	LOD: 0.1 pg. μL^-1^	0.1 ~ 100 pg μL^-1^	/	(Zhan et al., 2018)
	GNP/QD	Citrus Tristeza virus	LOD: 130 ng mL^-1^	0 ~ 1 μg mL^-1^	/	(Shojaei et al., 2016)
Crop Growth Sensors	Graphite/Carbon nanotubes	Fruit	GF was 48 at 50% strain and reached 352 for 150% strain	0 ~ 150%	/	(Tang et al., 2019)
	PANI	Stalks	GF was 3.8 at 10% strain	0 ~ 100%	/	(Borode et al., 2023)
	PVA-AC hydrogel	Stalks	GF was 1.006 at 50%, 1.667 at 200%, and 2.193 at 350%	0 ~ 350%	/	(Wang L. et al., 2023)
	PAA-RGO-PANI hydrogel	Fruits	GF = 4.5/4.58	0 ~ 200%	/	(Hsu et al., 2021)

### 3.1 Crop hormone sensors

Upon plant stress exposure, plant hormones function as pivotal signaling molecules, crucial for modulating plant metabolism and eliciting immune reactions (Meena et al., 2017; Waadt et al., 2022). Dynamic perception of endogenous hormone information in crops is crucial for the timely monitoring of crop growth status. The concentration of plant hormones within plants is very low and fluctuates with the state of the plant (Coatsworth et al., 2023). Thus, developing crop hormone sensors with high sensitivity, low detection limits, and good specificity is a significant challenge.

**Figure 4:**
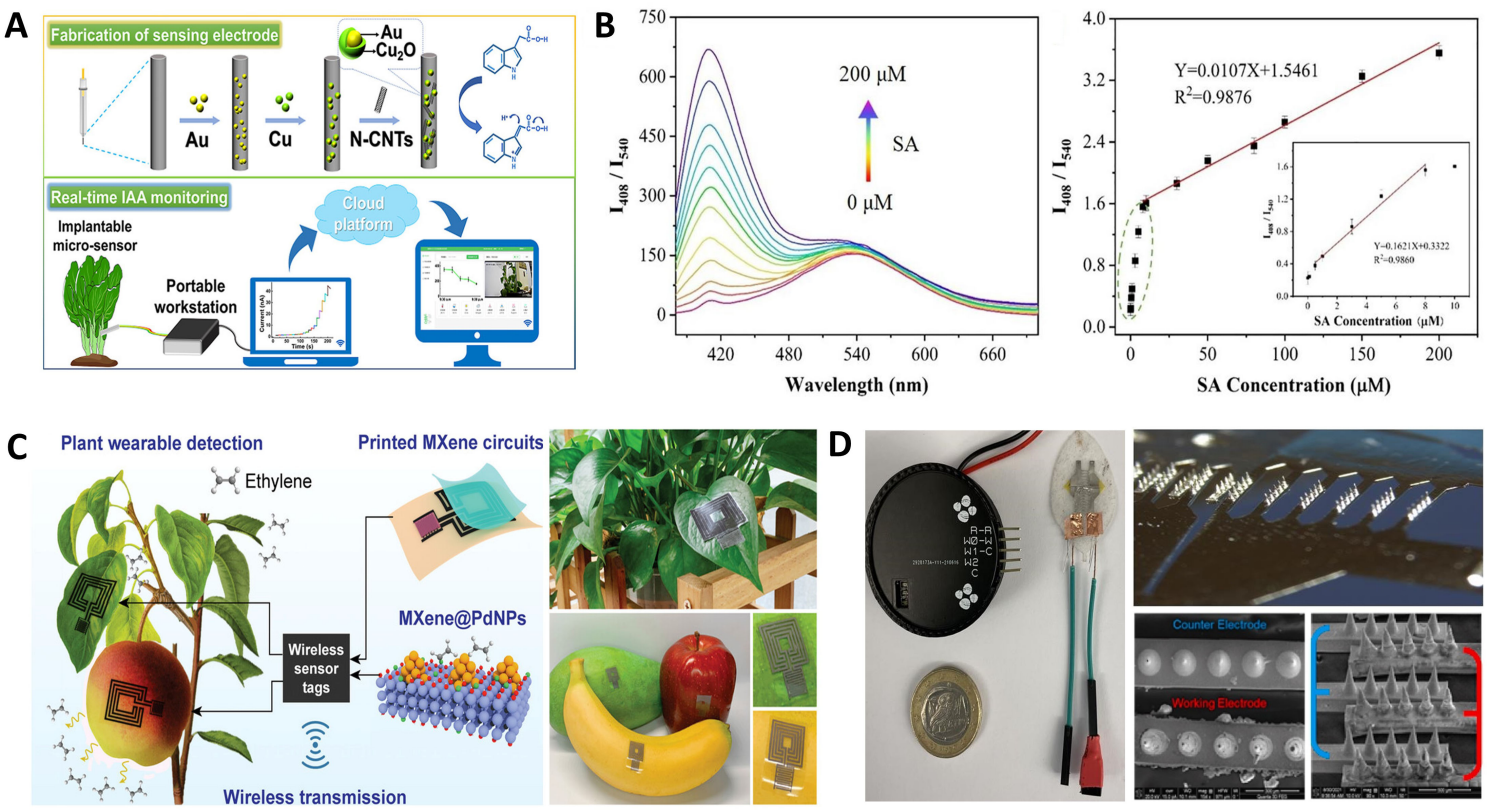
Crop hormone sensors. **(A)** An implantable microsensor system with the N-CNTs/Au@Cu_2_O/CFM electrode (Wu et al., 2022). **(B)** Detection performance of biomass-based fluorescent probes (Shi et al., 2024). **(C)** Mxene-based wireless sensor for ethylene monitoring (Li et al., 2023). **(D)** Interdigitated electrode-based microneedle sensor (Bukhamsin et al., 2022).

Many nanomaterials with unique physicochemical properties have been designed for crop hormone sensing, playing a significant role in improving the sensitivity, detection limit (LOD), linear detection range, response time, and reproducibility of electrochemical sensors. Wu et al. (2022) developed a miniature indole-3-acetic acid (IAA) sensor using a three-dimensional nanonetwork sensitive layer constructed by synergizing core-shell structured Au@Cu_2_O nanoparticles and nitrogen-doped carbon nanotubes. This IAA micro-sensor has a detection range of 1 to 10,000 ng/mL with ultra-low detection limits ranging from 10.8 to 57.8 pg/mL within a pH range of 4 to 8, suitable for real-time detection of IAA in living plants (**Figure 4A**). Furthermore, the realization of real-time monitoring necessitates the development of an intelligent analysis system capable of automatically acquiring and processing electrochemical data. The research team has devised an online intelligent analysis system grounded on the ANN (Artificial Neural Network) model, which encompasses reusable microsensors, a portable electrochemical workstation, a host computer, and an online cloud platform. This analytical system is capable of automatically interpreting raw data from electrochemical spectra and converting them into concentration information of IAA (Indole-3-Acetic Acid), thereby enabling intelligent processing and visualization of IAA information. Shi et al. (2024) developed a ratiometric fluorescent probe based on silk-derived carbon quantum dots@curcumin@iron metal-organic frameworks (SCQDs@Cur@Fe-MOFs), achieving sensitive detection of salicylic acid in rice with a detection limit as low as 0.14 μmol/L (**Figure 4B**). Li et al. (2023) fabricated a wireless wearable ethylene sensor based on a screen-printed MXene radio frequency resonator (**Figure 4C**). By introducing MXene@PdNPs to further modify the sensing element, the sensor achieved a more sensitive ethylene response and lower detection limits at room temperature, enabling *in situ* and continuous monitoring of ethylene content with a detection limit of approximately 0.084 ppm, and an operating range of 0.5 ~ 20 ppm. Notably, the flexible resonator, comprising a radio frequency antenna and a gas sensor, eliminates the need for intricate hardware and battery connections to each sensor, thereby facilitating information perception and wireless readout. The MXene-based platform has been successfully mounted on living plant organs, specifically four representative climacteric fruits, to monitor ethylene emissions *in situ*. This signifies the enormous potential of printed MXene electronics in the scalable manufacturing of wearable smart sensing devices for plants. However, high temperatures and humidity accelerate the degradation of sensitive materials, reducing the operational lifespan of sensors, limiting their application in harsh field environments. Future exploration of advanced packaging technologies for sensors could further enhance sensor stability. Currently, there remains a significant challenge and a vast knowledge gap in understanding the real-time dynamics of plants’ defensive responses under environmental stresses. An integrated wearable sensor suite tailored for crops has the potential to emerge as a pivotal tool in plant stress physiology research.

Traditional techniques for measuring hormone levels generally require destruction of large amounts of tissue to obtain necessary extracts, making it challenging to monitor crops in the field in real-time and sustainably. Therefore, it is necessary to develop highly sensitive, non-invasive sensing technologies to realize the potential of precision agriculture. Bukhamsin et al. (2022) proposed a wearable microneedle sensor for *in-situ* monitoring of salicylic acid (SA) in crops. The research team prepared microneedles with a height of approximately 240 μm on interdigital electrodes (**Figure 4D**) and functionalized the microneedle electrodes with a salicylic acid-selective magnetic molecularly imprinted polymer, achieving *in-situ* monitoring of salicylic acid in crops with a detection limit of 2.74 μM and a detection range up to 150 μM. Wang et al. (2021) proposed an abscisic acid (ABA) sensor based on Au@SnO-vertical graphene microneedle arrays, selecting vertical graphene (VG) films as an electrocatalyst and carrier for other catalysts, with Au@SnO_2_ nanoparticles as selective active sites for ABA. This sensor offers advantages such as small sensing volume, wide pH range, and low ABA detection limit (between 0.002 to 0.005 μM), enabling *in-situ* ABA detection in crops. It is worth noting that microneedles, as tools for extracting sensing substances, are susceptible to biological contamination. Continuous exploration of efficient antifouling strategies is needed to prevent the passivation of coating surfaces, thereby achieving long-term, accurate monitoring of crop hormones.

An intriguing and challenging area of applied research is the integration of microneedle sensing functions with microneedle drug delivery capabilities to develop a unified crop microneedle biosystem. This system would not only facilitate accurate monitoring of internal signaling molecules but also enable automated, precise delivery of agrochemicals based on monitoring results. Such advancements could significantly enhance precision agriculture practices and offer new tools for plant research and crop trait design, opening new avenues for applications in plant science and agriculture (Paul et al., 2019; Cao Y. et al., 2023).

### 3.2 Crop nutrient sensors

As the basis for crop growth and development, nutrients play an important role in maintaining normal physiological metabolism and improving yield and quality. Nutrient elements such as nitrogen, phosphorus, potassium, and zinc are closely related to crop growth, and the lack of any element may cause abnormal plant growth (Zhang et al., 2013). Traditional crop nutrient diagnosis requires destructive sampling in the field and biochemical analysis in the laboratory, consuming significant manpower and time. In recent years, biosensor technology and hyperspectral technology have rapidly developed as non-destructive means for crop growth monitoring. Biosensor technology offers unique precision advantages in monitoring the tissue or cellular level of crops. Currently, directly visualizing the spatiotemporal distribution of nitrogen nutrients within crops at the cellular level remains a major challenge. Sensors based on genetically encoded fluorescent proteins are powerful tools for studying dynamic nutrient distribution in crops, allowing minimally invasive monitoring of *in-situ* nutrient levels (Sadoine et al., 2023). Liu et al. (2022) developed the genetically encoded fluorescent nitrate sensor mCitrine-NLP7(NIN-LIKE PROTEIN 7), which specifically monitors the single-cell nitrate signal throughout seedlings. Chen et al. (2022) reported genetically encoded fluorescent biosensor NitraMeter3.0. This biosensor can real-time monitor NO_3_^−^ concentration at the cellular level, visualizing the spatiotemporal changes of NO_3_^−^ during the crop lifecycle. Lilay et al. (2021) proposed a zinc sensor based on the transcription factors bZIP19 and bZIP23, identifying the precise molecular mechanism of Zn sensing and transcriptional coordination in plants. Siqueira et al. (2020) used real-time fluorescence sensing technology, changes in nitrogen (N) and potassium (K) in crop canopies were detected during the early growth stages of maize (before the V6 growth stage). Giust et al. (2018) developed a portable sensor based on graphene oxide and upconversion nanoparticles (GO/UCNP), which evaluates the specific mRNA content of membrane transport proteins (ZIP) related to crop zinc (Zn) deficiency by monitoring different light output intensities, thus achieving early zinc deficiency assessment in crops. This sensor not only has good portability but also can be directly applied to the detection of RNA extracts, eliminating the extra steps required for reverse transcription or DNA amplification in RT-PCR.

### 3.3 Crop moisture sensors

Water plays a crucial role in the growth and development of crops. Drought stress-induced physiological changes in crops include restricted cell division and elongation, stomatal closure, reduced photosynthesis, and reduced yield (Melandri et al., 2020). Globally, drought stress causes more yield losses than any other singular biological or abiotic factor (Bhandari et al., 2023). The demand for high-performance moisture sensing in smart irrigation systems is rapidly increasing. However, achieving non-invasive, real-time, and precise tracking and monitoring of plant water status remains a significant challenge.

**Figure 5:**
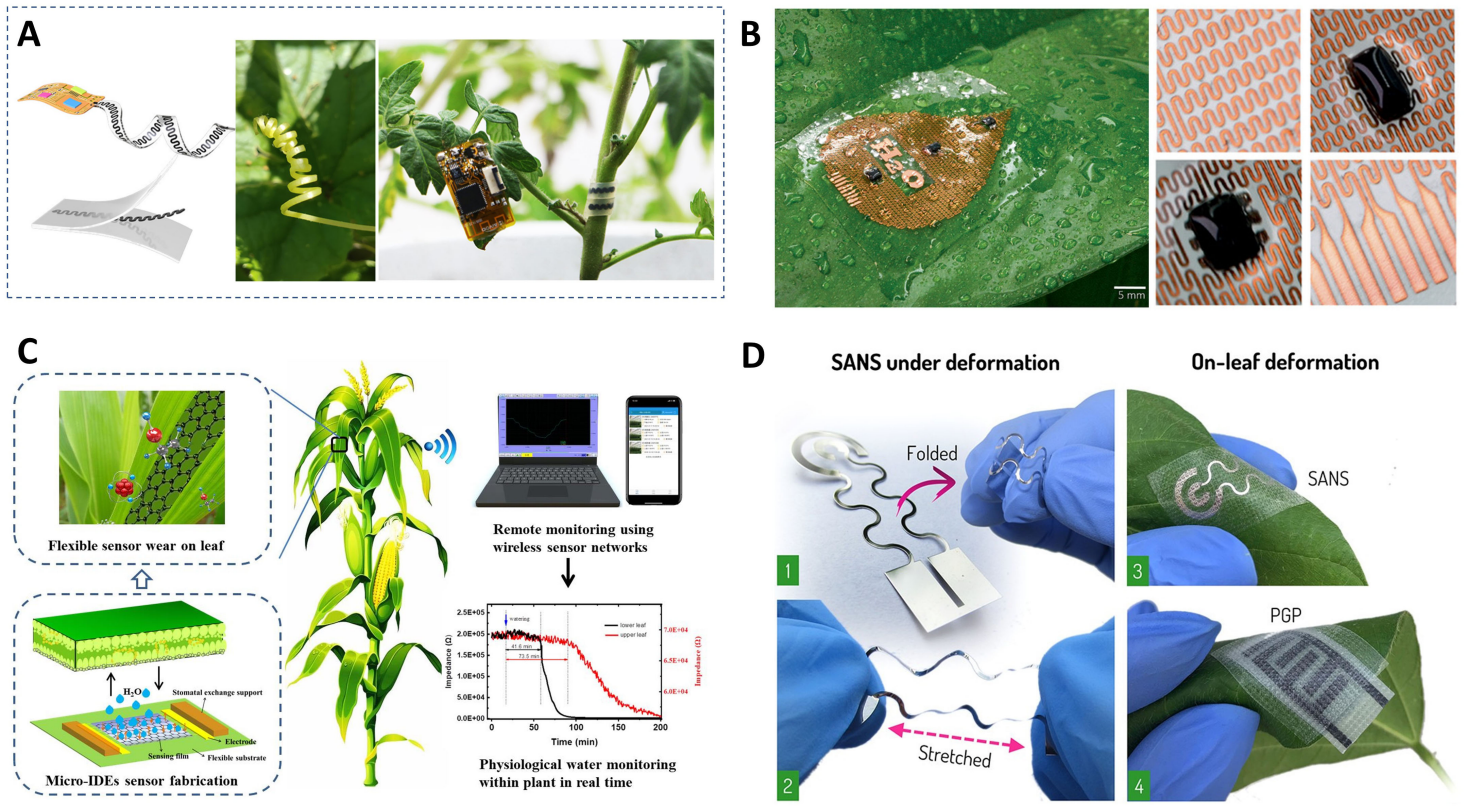
Crop moisture sensors. **(A)** adaptive winding strain sensor for plant pulse monitoring (Zhang C. et al., 2022). **(B)** Ultra-thin wearable flexible sensors for *in-situ* monitoring of water transport in crops (Chai et al., 2021). **(C)** Wearable crop moisture sensor based on nano-graphene oxide (Li D. et al., 2022). **(D)** Impedimetric flexible wearable sensors for on-site sensing of the water loss in plant leaves (Barbosa et al., 2022).

Crop flexible sensors can be attached to the crop surface to achieve rapid and accurate crop moisture sensing in a non-invasive manner. The sensing methods of crop water flexible sensors developed so far include plant stem pulse sensing, runoff heat transfer sensing, leaf water transpiration sensing, etc. Each of these methods has shown its own advantages. Inspired by adaptive coiling plant tendrils, Zhang et al. (2022) developed an integrated crop wearable system (IPWS) based on an adaptive winding strain (AWS) sensor (**Figure 5A**). The IPWS can monitor the expansion and shrink of plant stem wirelessly and reflect the growth and water state of tomato in real time. With the serpentine-patterned laser-induced graphene, the AWS sensor exhibits excellent resistance to temperature interference with a temperature resistance coefficient of 0.17/°C. The IPWS comprises three modules: the AWS sensor, the flexible printed circuit, and the smartphone APP display interface, which collectively enable the wireless transmission of resistance variation data to a smartphone for recording purposes. Chai et al. (2021) reported the first flexible plant stem flow wearable sensor (**Figure 5B**). The primary sensing components are two aligned temperature sensors with a positive temperature coefficient (PTC) thermistor in the middle. When there is a sap flow in the stem, an anisotropic temperature distribution occurs, which can be monitored by the two temperature sensors, thereby allowing the analysis of the sap flow rate. This sensor is ultra-thin, soft, and stretchable, with a thickness of only 0.01 mm, featuring excellent water/air/light permeability. It can continuously and non-invasively monitor the dynamic transmission and distribution process of water within herbaceous plants in real time without affecting plant growth. To demonstrate the application ability of the sensor in the farmland, the watermelon stem sap flow was continuously measured for 18 hours in the farmland, and the data was transmitted through a wireless control system developed for remote control and data acquisition. Remote control (on/off), wireless data acquisition, and graphical display of sensors are achieved through a customized smartphone application. The sensors can be successfully used in small herbaceous plants in a continuous and non-destructive manner, providing experimental support for the practical application. Different from the above monitoring methods, Li et al. (2022) developed a wearable crop leaf moisture sensor based on flexible graphene oxide (GO) (**Figure 5C**), achieving real-time on-site sensing of plant transpiration. By deploying multiple sensors on different leaves of the plant or different parts of the same leaf, the internal water transport within the plant can be dynamically monitored. Combined with a photosynthesis monitoring system, the responses and synergistic effects of crop net photosynthetic rate and transpiration under different light environments could be revealed. Because the sensors are installed on the lower surface of the leaf, it does not interfere with the physiological functions of the plant. The sensor provides a new technical method to carry out quantitative monitoring of crop water in the entire life cycle and build smart irrigation systems. Impedimetric wearable sensors are also a promising strategy for determining leave water because they can afford on-site and nondestructive quantification of cellular water. The sensor has a sensitivity of 7945 Ω/%RH, an operating range of 11 ~ 95% RH, and a response time of 20.3s. Barbosa et al. (2022) developed high-adhesion impedance-based wearable sensors for monitoring leaf water content (LWC), allowing for the long-term monitoring of LWC in soybean leaves (**Figure 5D**). Stand-alone Ni structures (SANS) were fabricated through photolithography and electroplating steps, composed of two pads interconnected by serpentine lines to form a stretchable structure, further promoted for high adhesion to the leaf using typical adhesive tapes. This wearable sensing electrode showed strong adhesion to the underlying furry leaf surfaces without delaminating or cracking upon bending. The sensor has a sensitivity of 27.0 kΩ %^-1^, an operating range of 0-70% and 70-90% LWC, and a response time of 2 s. There are many other new monitoring methods that can perform non-destructive monitoring of crop moisture. For example, Yin et al. (2021) deployed a wearable leaf VPD sensor. This device used a flexible polyimide sheet as the substrate, measuring relative humidity (RH) with laser-induced graphene (LIG) sheets and temperature with gold (Au)-based thin-film thermistors. Results showed that patterned LIG responded swiftly to RH changes caused by transpiration, and combined with integrated thermistors, it enabled continuous monitoring of VPD on leaves for over 2 weeks. Moreover, the wearable VPD sensors have been deployed in farmland. The sensor can differentiate transpiration between fertilized and unfertilized corn plants. It was found that fertilized plants release more water vapor into the air due to their higher levels of photosynthesis and transpiration. Graphene-based capacitive humidity sensors have also demonstrated rapid responses to RH changes (Zhao et al., 2020). Recently, Cecilia et al. (2022) developed a non-invasive online crop leaf moisture sensor based on the attenuation of photons passing through the leaf, the signals recorded by the sensor showed good consistency with destructive measurements across all species, enabling continuous and reliable monitoring of leaf moisture conditions.

### 3.4 Crop disease monitoring sensors

Plant diseases have a significant impact on the quality and yield of plants, and if not detected early and controlled in time, they can cause substantial losses. Direct losses caused by pathogens account for about 10% to 15% of global agricultural productivity (Mohammad-Razdari et al., 2022). Traditional detection of plant pathogens, bacteria, viruses, etc., mainly relies on microbiological or PCR-based techniques (Schena et al., 2008; Khiyami et al., 2014), which require relatively large amounts of target substances and depend on multiple detections to accurately identify different plant pathogens. Therefore, they lack portability, are time-consuming, and require professional personnel. Methods such as thermal imaging, hyperspectral technology, and fluorescence imaging detect pathogens by analyzing their impact on plants, such as changes in plant morphology, transpiration rate, and temperature. However, these methods have high equipment costs and lack precision and convenience (Fang and Ramasamy, 2015; Martinelli et al., 2015). In recent years, new sensing technologies such as electronic noses, wearable sensors and “electronic eyes” have attracted increasing attention in the field of crop disease monitoring due to their high sensitivity and portability. Electronic nose systems can detect characteristic volatile organic compounds (VOCs) released by crop tissues and are used for crop health diagnostics. Rapid analysis of characteristic VOCs, as a potential non-invasive technique, can be used for early diagnosis of crop diseases (Li et al., 2019). Li et al. (2021a) developed a VOC sensor that can be installed on leaves for real-time fingerprint identification of plant volatile organic compounds. This invisible sensor patch integrates graphene-based sensing material arrays and flexible silver nanowire electrodes on a stretchable substrate, accurately detecting and classifying 13 key plant volatile VOCs with a classification accuracy of over 97% and detection limits as low as low ppm or sub-ppm levels. This allows for early diagnosis of tomato late blight infection (within 4 days after inoculation) and abiotic stress such as mechanical damage (within 1 hour). Nanosensors, with their high sensitivity and precision, have become an important new tool for crop disease assessment. Nanoscale electronic noses also have the ability to sensitively distinguish between plants infected with different pathogens. Greenshields et al. (2016) used a nanoscale electronic nose to detect strawberries infected with Aspergillus niger, Rhizopus, and uninfected strawberries. The use of nanotechnology combined with biosensors to prepare bionanosensors can significantly shorten detection times, indicating the presence of crop diseases (Kwak et al., 2017). Freitas et al. (2019) proposed a low-cost, rapid magnetic immunoassay method for ultra-sensitive detection of the coat protein (CP-CTV) of the citrus tristeza virus. The constructed immunosensor showed good linearity, with a wide linear concentration range of 1.95-10.0 × 10^3 fg mL^-1^ and an ultra-low detection limit of 0.3 fg mL^-1^. In addition, optical sensing methods such as colorimetry, fluorescence, and surface plasmon resonance (SPR) can also monitor plant health and will not be elaborated further here. It should be noted that, considering the common shortcomings of existing technologies, advancements in nanomaterials and new biomarkers will provide superior performance solutions for plant health monitoring.

### 3.5 Crop growth sensors

Crop growth is a highly dynamic process with daily micro-variations. Accurately monitoring these daily micro-variations is the foundation for timely and precise water and fertilizer operations, and even for understanding crop growth mechanisms, which is crucial for ensuring optimal yield and sustainable resource utilization (Wuyts et al., 2015; Cosgrove, 2018). High-sensitivity and high-resolution detection tools for monitoring micro-deformation in crop growth pose significant challenges. In recent years, flexible and stretchable sensors have garnered significant attention, researchers have developed various flexible wearable sensors by combining wearable technology with advanced functional materials such as nanomaterials and conductive hydrogels. For instance, Tang et al. (2019) utilized the synergistic enhancement between graphite and carbon nanotube films to prepare a flexible, stretchable (strain rate of 150%) carbon nanotube/graphite resistive strain sensor by depositing graphite and carbon nanotube conductive ink on disposable latex glove sheets. This successfully monitored crop growth from nanometers to centimeters, revealing rhythmic growth patterns in Solanum melongena L. (eggplant) and Cucurbita pepo (zucchini) fruits. This carbon-based sensor boasts low manufacturing costs and high sensitivity. However, the stretchability of carbon based sensors may be limited as they are prone to fracture under high levels of tension. The various sizes and shapes of different plant organs present challenges for the design and manufacture of wearable strain sensors. 3D printing technology, with its flexible manufacturing processes, allows easy alteration of the size and shape of the sensor band, making it applicable in strain sensor manufacturing. Lee et al. (2022) have developed a liquid polymer/metal salt-based stretchable strain sensor based on polyethylene glycol (PEG) and silver nitrate composites using 3D printing technology. The researchers used this sensor to monitor the shape and size changes of citrus and passion fruit continuously for one week, demonstrating excellent stability, extensibility (strain rate of 30%), and linear resistance change coefficient (R^2^>0.99). Borode et al. (2023) developed and applied a new low-cost polyaniline (PANI)-based strain sensor to monitor plant growth. This sensor was made by *in-situ* chemical polymerization of aniline on an elastic band substrate via dip-coating. PANI nanoparticles adhered to the substrate fibers and their gaps, giving the sensor good conductivity, and it was successfully applied to monitor the growth of sunflowers and soybeans. Continuous monitoring of crop deformation requires strain sensors to have fatigue resistance and high stability; otherwise, the monitoring results may be distorted or require frequent calibration, causing inconvenience in practical applications. Wang L. et al. (2023) developed a new conductive hydrogel strain sensor based on a high-viscosity polyvinyl alcohol (PVA) solution and conductive activated carbon (AC). This sensor was formed by uniformly dispersing conductive activated carbon (AC) in a high-viscosity polyvinyl alcohol (PVA) solution to create a continuous conductive network and was simply prepared through freeze-thaw cycles. The prepared strain sensor exhibited low hysteresis (<1.5%), fatigue resistance (fatigue threshold of 40.87 J m^-2^), high working range (0 ~ 350%), and long-term sensing stability under mechanical cycles, monitoring plant growth for 14 days. Long-term automatic monitoring of sensors also requires a sustainable power supply. Energy supply for electronic devices in complex agricultural natural environments is a challenging task. Hsu et al. (2021) designed and developed a self-powered multifunctional wearable sensor for crop growth with a GF of 4.5/4.58 and an operating range of 0% to 200%. The sensor not only has excellent stretchability (650%) and mechanical stress strength (1050 Kpa), making it suitable for plant wearable sensors for plant growth but also can collect clean energy from dynamic sources such as wind and rain and store the energy.

## 4 Multimodal sensors for monitoring crop habitat information

### 4.1 Microclimate sensors

**Figure 6:**
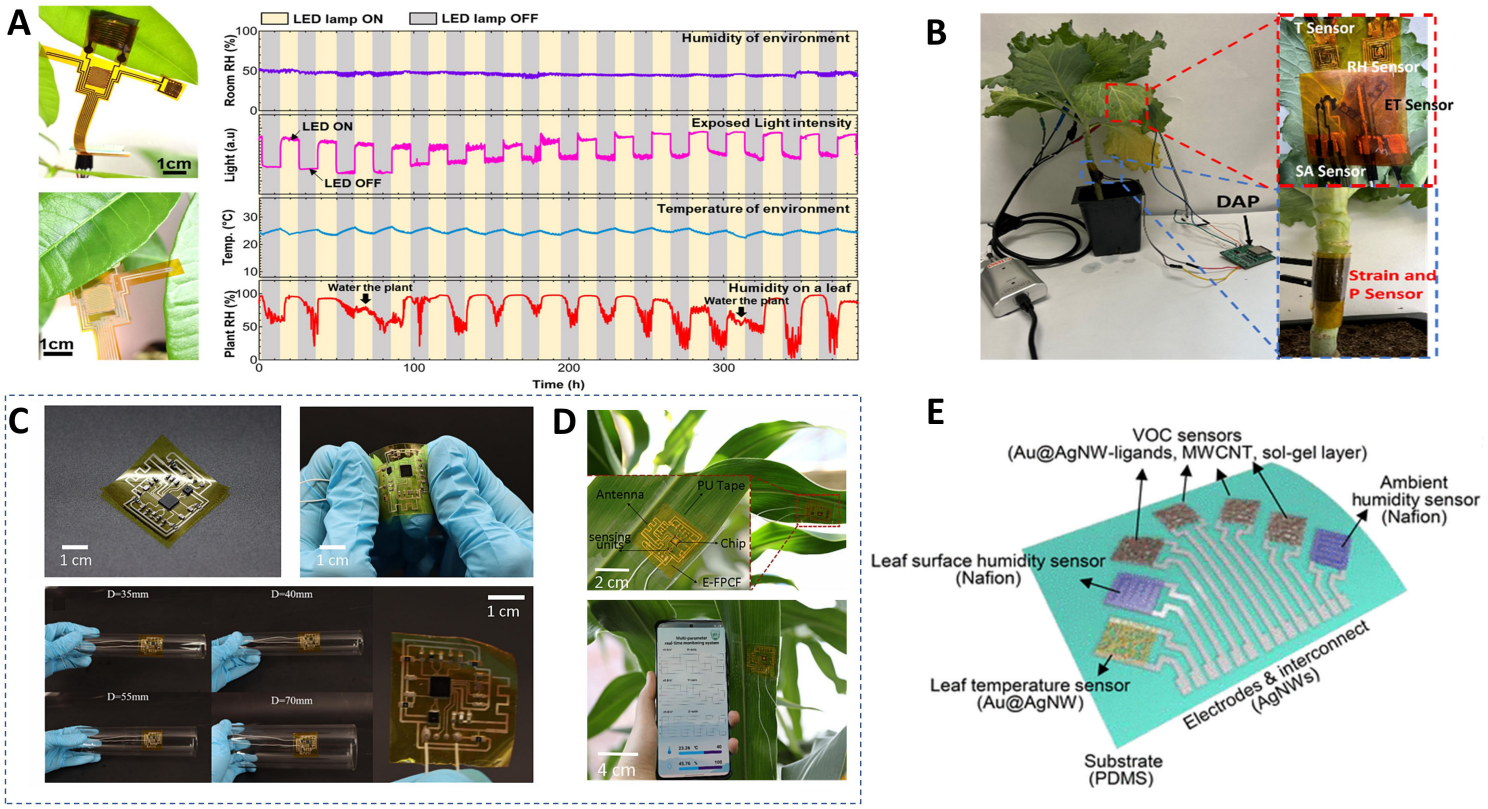
Crop growth microclimate sensors. **(A)** The image on the left shows the multimodal flexible sensor device mounted on the underside of the leaf, and the image on the right shows the monitoring results (Lu et al., 2020). **(B)** The optical image of hybrid multifunctional physicochemical sensor suite installed on a cabbage plant (Hossain and Tabassum, 2023). **(C, D)**. Semi-embedded flexible multifunctional sensor for on-site continuous monitoring of plant microclimate (Qu et al., 2024a). **(E)** Overview of the multimodal wearable sensor for continuous plant physiology monitoring (Lee et al., 2023).

The complex relationship between the microenvironment in which crops grow and their yield underscores the importance of real-time and multi-parameter monitoring of the environment (Hackenberger et al., 2018). While traditional crop environmental sensors have been constrained to measuring a single ecological parameter, advancements in the miniaturization and integration of sensors in recent years have made multi-parameter monitoring a feasible reality. Zhao et al. (2019) developed a multifunctional stretchable leaf-mounted sensor. This sensor, with dimensions of 13,000 × 8,800 × 30 μm and a weight of only 17 mg, incorporates several heterogeneous sensing elements made of metals, carbon nanotube matrices, and silicon, enabling the detection of temperature, moisture, light intensity, and strain in leaves. They attached sensors to corn leaves for preliminary outdoor experiments, demonstrating the sensors’ versatile monitoring capabilities under real-world conditions. Additionally, the sensor can operate in networks with a node-to-node distance of at least 200 meters, which is ideal for open-field environments in precision agriculture applications. Lu et al. (2020) used stacked ZnIn_2_S_4_ (ZIS) nanosheets as the sensing medium and integrated humidity, light, and temperature sensors on a flexible PI substrate (50 μm thick), designing a highly integrated multimodal flexible sensor system that realizes the monitoring of microenvironments and crop health status (**Figure 6A**). The multimodal flexible sensor device was connected to a data logger which was interfaced with a laptop computer. Users could directly observe the sensing results on the laptop. Hossain and Tabassum (2023) pioneered a wearable physicochemical sensor kit “PlantFit” (**Figure 6B**) using low-cost and roll-to-roll screen printing technology, designed to simultaneously measure two key plant hormones, salicylic acid and ethylene, as well as the vapor pressure deficit and radial growth of the stem in living plants. The sensor can record environmental temperature and humidity data, along with data on salicylic acid content, ethylene content, and stem diameter changes in bell peppers continuously for 40 days. Qu et al. (2024a) applied a unique semi-embedded design method and biocompatible materials to fabricate a semi-embedded flexible printed circuit film (E-FPCF) connecting microchips to various heterogeneous sensing elements, developing an ultra-thin sensor with a conductive layer thickness of only 7 microns (**Figures 6C, D**). By connecting the micro-chip to various heterogeneous sensing elements, parallel, multiple, and continuous monitoring of plant surface temperature and humidity is possible. Real-time display and storage of microclimate data can be accomplished through mobile applications. Lee et al. (2023) integrated a multifunctional wearable crop sensor (**Figure 6E**) with a machine learning model. By using an algorithmic model to analyze and process multi-channel real-time sensor data, they achieved continuous monitoring of crop volatile organic compounds (VOCs) and microenvironmental temperature and humidity, and quantitatively detected Tomato spotted wilt virus on the fourth day post-inoculation. This provided useful insights for researching intelligent and automated crop growth microenvironment sensing systems.

In multimodal sensors, the presence of multiple stimuli can lead to cross-interference effects, impacting the sensor’s accuracy in detecting specific targets. The complexity of interface parameters imposes higher demands on the functionality of sensing materials and the richness of sensing mechanisms. Therefore, several challenges deserve attention in future sensor design and development: first, exploring strategies for decoupling sensing mechanisms (such as through different sensing materials, sensor layouts, and signal processing methods); second, implementing temperature drift compensation to ensure thermal stability of the system; and third, developing appropriate module encapsulation to minimize interference from environmental factors (such as gases and humidity), while not affecting and potentially improving the inherent performance and cyclic stability of the module.

### 4.2 Soil sensors

Soil is a crucial production factor for crop cultivation. The quality of soil is primarily determined by parameters such as soil moisture content, soil nutrient content, soil pH, and soil pollutants (Yin et al., 2021). In pursuit of a comprehensive and precise evaluation of soil’s physical properties, chemical composition, and biological activities, researchers have relentlessly explored and innovated by integrating diverse sensors and technological approaches to conduct omnidirectional and multi-faceted monitoring of soil conditions. **Table 2** systematically showcases the sensing mechanisms, measurement accuracies, and measurement ranges of current mainstream soil sensors. Each of these sensors possesses unique strengths, enabling them to precisely capture various critical information within the soil. When integrated into a multimodal monitoring system, these sensors collaborate seamlessly, jointly mapping out a detailed soil health profile that offers agricultural producers unprecedented decision-making insights.

**Table 2:** Soil sensors.

Application	Sensing Mechanisms/ Sensitive Materials	Measurement Accuracy	Working Range	Reference
Soil Nutrient Sensors	Potentiometric sensing/Thick film electrodes for AuPt	LOD: 291 nM	1 ~ 10 μM	(Ramesh et al., 2023)
	Potentiometric sensing/Polymeric membranes	Sensitivity: -47 ± 4.1 mV/dec	0.05 ~ 100 mM	(Baumbauer et al., 2022)
	Hydrogel-Coated potentiometric solid-state ion-selective membrane	Sensitivity: 43.04 mV/dec	/	(Fan et al., 2022)
	Impedance sensing/Graphene interdigital electrodes	LOD: 1.71 ppm	1 ~ 160 ppm	(Ludena-Choez et al., 2022)
Soil Moisture Sensors	Capacitive sensing/Cr-Soc-MOF-1 coating	Sensitivity:∼450% on clayey soil	2 ~ 40% RH	(Alsadun et al., 2023)
	Capacitive sensing/MoS_2_ Nanosheets	Sensing response (~43684%)	11 ~ 96% RH	(Siddiqui et al., 2022)
	Resistive sensing/Graphene-carbon (G-C) ink-based sensor	Sensitivity: ∼12.4 Ω/%RH	25 ~ 91.7%RH	(Beniwal et al., 2023)
Soil pH Sensors	Potentiometric sensing/Ion selective electrodes	Sensitivity: -61.05 mV/pH	3 ~ 11 pH	(Mccole et al., 2023)
	Voltage sensing/antimony all-solid-state electrodes	Sensitivity: -38.2 mV/pH	3 ~ 8 pH	(Nair et al., 2022)
	Graphene-carbon (G-C) modified zinc oxide (ZnO)-based active layer	Sensitivity: 5.27KΩ/pH	2 ~ 8 pH	(Aliyana et al., 2022)
	Current sensing/Graphene/L-Arginine	Sensitivity: 97μS/pH	3 ~ 10 pH	(Siddiqui and Aslam, 2023)
Soil Heavy Metal Sensors	Current sensing/rGO/g-C_3_N_4_	The LOD for both Cd and Zn were 0.01 mg kg^-1^.	Cd: 20 ~ 450 mg/L Zn: 20 ~ 1100 mg/L	(Eleney et al., 2020)
	Voltage sensing/Polyaniline inks	The LOD of Cu^2+^, Pb^2+^, and Hg^2+^ were 55.4 pM, 22 pM, and 17.8 pM, respectively	Cu^2+^: 0.2 ~ 1500 nM Pb^2+^: 0.2 ~ 100 nM Hg^2+^: 0.1 ~ 10 nM	(Zhao et al., 2020)
	Photoelectric sensor/QR-CDs/EDTA-Tb^3+^	Identify Cr^6+^, Fe^2+^, Cu^2+^, Fe^3+^, Mn^2+^, Co^2+^, Ni^2+^ at 0.05μM	0.05 ~ 50 μM	(Xu et al., 2022)
	Microbial fuel cell (SMFC) sensor	The LOD of Cu^2+^ were 1 mg L^-1^	12.5 ~ 400 mg/L	(Liu et al., 2020)

**Figure 7:**
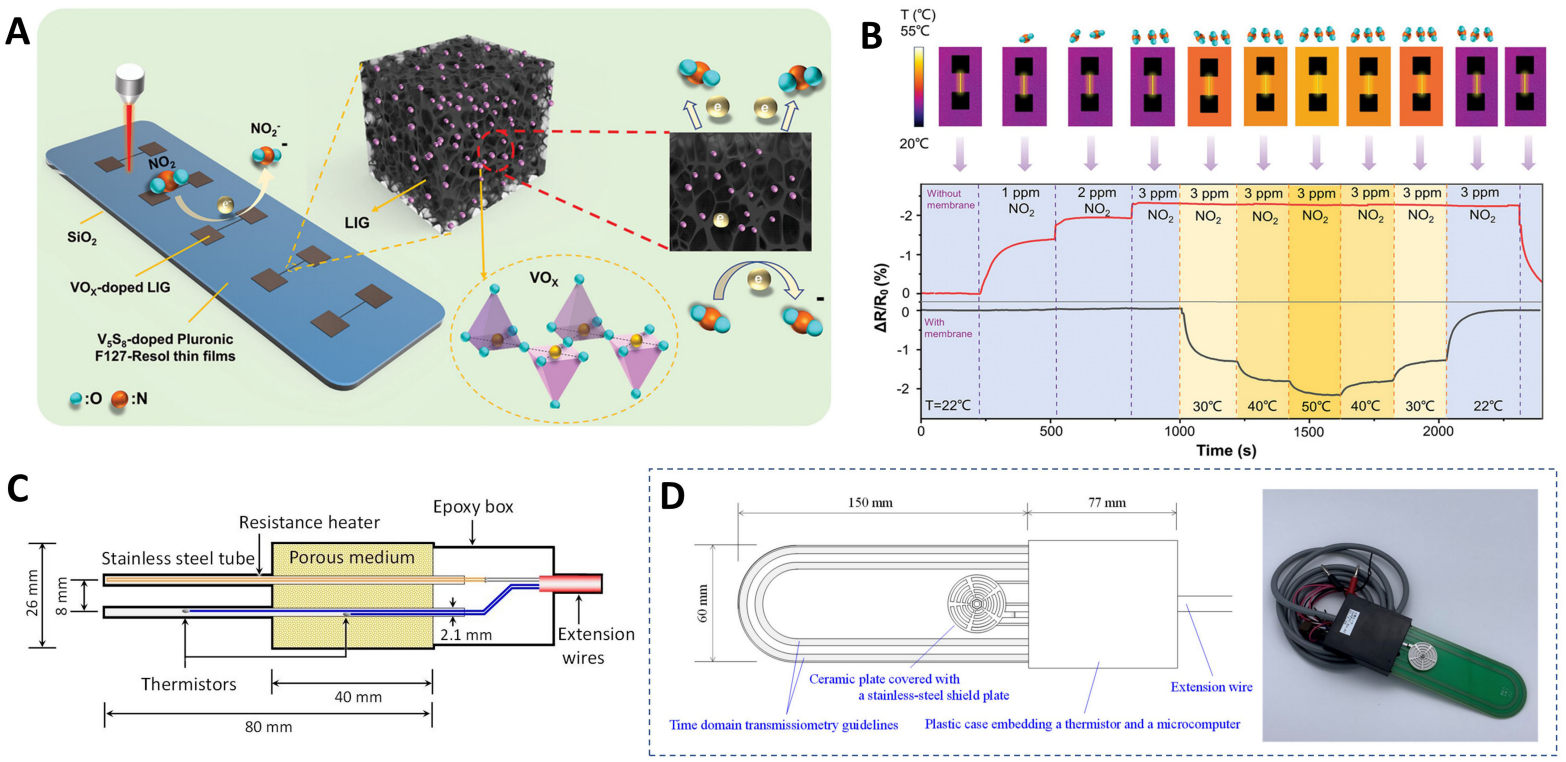
Soil sensors. **(A)** Principle and structure of vanadium oxide-doped Laser-Induced graphene multi-parameter sensor (Yang et al., 2023). **(B)** Application of the encapsulated VOX-doped LIG sensor (yellow shaded region) and the unencapsulated one operated at 50°C from self-heating to completely decouple NO_2_ gas and temperature. Encapsulated sensors can accurately detect temperature changes (Yang et al., 2023). **(C)** The DPHP sensor that simultaneously measures soil thermal properties, water content and matric potential (Kojima et al., 2021a). **(D)** TDT - based sensor for simultaneously measuring soil water content, electrical conductivity, temperature, and matric potential (Kojima et al., 2023b).

Within multimodal sensors, the concurrent monitoring and processing of multiple, disparate physical quantities or signals are indispensable, yet these signals are prone to mutual interference or coupling. Hence, the decoupling mechanism is indeed of paramount significance. Yang et al. (2023) have showcased a proof-of-concept demonstration for a multi-parameter decoupled sensor. They developed a high-performance multi-parameter sensor based on vanadium oxide (VOX)-doped laser-induced graphene (LIG) foam to completely decouple nitrogen oxides (NOX) and temperature (**Figure 7A**). The encapsulation of the sensor with a soft membrane further allows for temperature sensing without being affected by NOX. The unencapsulated sensor operated at elevated temperature removes the influences of relative humidity and temperature variations for accurate NOX measurements (**Figure 7B**). The heterojunction formed at the LIG/VOX interface endows the sensor with an enhanced response to NOX and an ultralow limit of detection of 3 ppb (theoretical estimate of 451 ppt) at room temperature. Concurrently, the sensor is capable of accurately detecting temperature over a wide linear range of 10 - 110°C. Integrating sensors with data processing and wireless transmission modules can further establish a remote environmental monitoring system that detects NOx and temperatures. Yue et al. (2024) developed a high-performance biochar nano-enzyme sensor array based on the principle of different effects of pesticides on the peroxidase-like activity of biochar. The research outcomes demonstrate that the five pesticides significantly suppress the peroxidase-mimetic activity of SP-BC, CH-BC, and EP-BC to varying degrees, presenting a solid basis for the realization of multimodal monitoring strategies. This sensor successfully distinguished and detected five pesticides in soil at concentrations ranging from 1 to 500 μM.

The dual-probe heat pulse (DPHP) sensor has been extensively employed for the determination of soil thermal properties. Integrating DPHP with other technologies represents an effective approach to achieving multi-parameter monitoring of soil properties. Kojima et al. (2021a) proposed a novel sensor based on the DPHP technique. The sensor features a heating wire that concurrently supplies thermal energy to both the soil and the sensor porous medium (**Figure 7C**). This sensor has been successfully employed to measure soil thermal properties, soil water content (ψ), and soil water matric potential (θ). The effective range of ψ measurements was -1000 to -2.5 m of water, and the accuracy of the ψ measurements was particularly good in the range of -350 to -2.5 m of water. Kojima et al. (2023b) proposed a novel sensor based on time domain transmissiometry (TDT), which can simultaneously measure soil water content (θ), matrix potential (ψ), electrical conductivity (σ_b_), and temperature (**Figure 7D**). Laboratory tests have demonstrated that this sensor exhibits low temperature dependence and long service life, enabling the simultaneous determination of θ and ψ, thereby providing vital information for *in-situ* soil water retention curves.

The emergence of multimodal data monitoring has empowered agricultural managers to concurrently capture and analyze information from diverse sources, fostering a deeper comprehension of both the inherent mechanisms and external manifestations of agricultural systems. However, in practical agricultural production, the deployment of multimodal sensors encounters several challenges. Firstly, compatibility issues arise from differences among sensors, particularly in terms of electrode types, physical interfaces, and communication protocols. These differences hinder the level of integration and miniaturization that can be achieved with multimodal sensors. Secondly, the decoupling of multi-parameters presents a significant challenge. For instance, a single sensing material may be sensitive to both temperature and humidity, potentially leading to interferences or coupling effects between these parameters. Accurate decoupling of these parameters is essential to obtain precise crop growth information, which is a critical issue to address when implementing integrated multi-sensor systems. Furthermore, the dynamic variability of agricultural environments can affect sensor measurements through various objective factors. In order to ensure that the collected data accurately reflects the growth status of crops, it is necessary to adopt adaptive compensation technology to adjust sensor parameters and measurement algorithms in real time according to environmental changes.

## 5 Challenges, future perspectives and summary

In recent years, the development of agricultural sensors has been significantly advanced by numerous technologies, leading to the continuous emergence of novel sensor designs. However, a substantial portion of related research remains in its infancy. Sensors encounter numerous challenges regarding stability, multi-functional integration, and cost-effective manufacturing, thereby hindering their adaptability to the demanding working conditions of large-scale agricultural fields. Although research progress has been made in agricultural smart sensors and they have shown great potential, compared with the industrial and medical fields, the application of smart sensors for plant monitoring is lagging behind and requires technological improvements. Existing smart sensor systems for plants need to overcome some obstacles. This section will describe the challenges that need to be overcome at present and the promising development directions for the future.

### 5.1 Challenges

#### 5.1.1 Robustness and long-term monitoring stability of sensors

At present, most studies are conducted in controlled environments such as laboratories and greenhouses, where sensors are fixed to plant leaf organs with adhesive tape or directly implanted into stems for short-term monitoring. The harsh and complex environment of agricultural planting scenarios, such as cold and heat, strong light, humidity, and storms, requires sensors to have long-term stability under extreme conditions. However, harsh environments may lead to melting of coating materials, changes in the internal stress structure of sensing layer materials, and drift of sensing signals in the sensing materials during long-term monitoring. In addition, the physiological effects of plants and environmental changes may also cause the adhesive tape to fail, resulting in loosening of the combination of sensors and monitoring points. To overcome this technical challenge, it is necessary to develop new materials to ensure the sensing stability of sensor materials in such harsh environments. In addition, it is necessary to ensure the strong adhesion and high biocompatibility of sensors with plant monitoring points, while maintaining minimal invasiveness. There are several ideas that may overcome the above challenges. First, develop wearable sensing devices that can be directly and firmly printed on plant organs, such as on the lower epidermis of plant leaves and stems, without affecting the normal physiological processes of photosynthesis and transpiration in plants. Secondly, the packaging of sensors is crucial, and further research is needed in the future to develop highly sealed materials and protective covers to minimize or eliminate the impact of external environments on sensors. In addition, it is necessary to study intelligent decoupling algorithms that can decouple the effects of environmental changes or plant physiological processes on sensor output changes, and optimize the self-calibration mechanism of sensors, to achieve high stability even in the constantly changing climate environment of farmland.

#### 5.1.2 Environmental impact and toxicity of new sensing materials

Considering the emerging sensors such as nanosensors and wearable devices are applied to some new materials like nanoparticles, two-dimensional nanomaterials, fluorescent quantum dots, biomaterials, and so on, it is a challenge that must be overcome to clarify and reduce or even eliminate the potential adverse effects of sensor materials on crop plants and the environment. Before large-scale deployment of these sensors, it is necessary to study the physiological and biochemical impacts and toxicity of these materials on crops in more detail, and also to assess their penetration, absorption, distribution, and fate in plant organs, ensuring that the deployment of these sensors does not affect plant vitality nor cause environmental and agricultural product pollution while maximizing volatilization potential. Not only is it necessary to assess whether the concentration of materials is hazardous, but also to evaluate factors such as the size, surface area, surface charge, morphology, surface chemistry, and aggregation of materials, all of which are related to their toxicity levels (Paramo et al., 2020). At present, there have been many studies on the toxicity of nanomaterials to plants, but most are studies of short-term effects, and research on the long-term impact on the complex ecological system of agriculture is still relatively lacking. Therefore, a large amount of research and empirical work still needs to be carried out, and relevant safety standards and regulations need to be developed. Only by overcoming these challenges can the large-scale successful application of new sensor materials in agriculture be possibly realized.

#### 5.1.3 Cost-effectiveness and user affordability of high-performance sensors

At present, the high cost of some raw materials and the cost of the production process have adversely affected the commercial application of high-performance sensors. In order to promote the large-scale application of sensors in agriculture, more attention should be paid to reducing the overall cost of sensors, such as developing mass-produced, low-cost preparation processes, and improving their durability in harsh agricultural environments to reduce replacement and maintenance costs. In addition, further research can be conducted on the integration of self-powered technology and low-power wireless sensor network technology. This integration will enable sensors to operate independently of external power sources, thereby further reducing operating and maintenance costs. Given the vastness and diversity of agricultural applications, the standardization and modular design of agricultural sensors is of crucial importance. This approach will facilitate the reduction of sensor production costs and enhance their cost-effectiveness in practical agricultural applications. Developing sensors with excellent versatility and adaptability, alongside the establishment of standardized interfaces and communication protocols, will facilitate their seamless integration into existing agricultural management systems, enabling more intelligent monitoring and decision support. For instance, when integrated with intelligent devices such as drones and robots, sensors can be utilized for various purposes, including crop monitoring, pest and disease early warning, and soil management, thereby enhancing their scalability in agricultural applications. To achieve this, standardized communication protocols and interoperability among different sensor types and systems are indispensable.

### 5.2 Future perspectives

Through a comprehensive review, we believe that the future development of new advanced sensors for intelligent farming mainly relies on the following aspects:

#### 5.2.1 Innovative multi-modal sensor arrays for enhanced monitoring and parameter decoupling

Sensors are extensions of human senses, and human perception systems can simultaneously sense and process different types of information in a very small perception field and complex environments. This also means that ideal agricultural sensors will consist of sensor arrays with multiplexing functions, capable of monitoring various biomarkers or parameters in the agricultural environment to evaluate agricultural production conditions from a more objective and comprehensive perspective. With the integration of advanced manufacturing technologies such as MEMS, micro-nano fabrication, and 3D printing in the agricultural sensor field, innovative development of light, thin, compact, and integrated multi-modal, arrayed advanced agricultural sensors will become a research hotspot. However, when multiple target input signals are present simultaneously, cross-interference may reduce monitoring accuracy (Yang et al., 2022). How to reduce signal crosstalk and cross-sensitivity characteristics between multiple parameters remains a challenge for current multi-modal sensors. In the future, with materials that have different response times to different input stimuli; employing different sensing mechanisms (e.g., current and resistance); exploring multi-modal sensing array unit layouts with decoupling properties, efficient multi-modal signal decoupling can be promoted to construct truly multi-modal sensors.

#### 5.2.2 Advanced sensor edge computing for efficient data analytics

With the rapid development of smart agriculture, the number of agricultural sensor nodes is continuously increasing at a high rate. It is necessary to move some computing tasks from cloud computing centers to edge devices to reduce the redundant data transmission between sensor terminals and computing units, thereby reducing the energy consumption of the sensing system. Sensor edge computing provides new possibilities for improving the efficiency of sensing systems (Ullo and Sinha, 2020; Zhou and Chai, 2020). In the traditional sensing-computing architecture, sensors and computing units are placed separately, and data conversion-based transmission leads to inefficient transmission and high latency. In the sensor edge computing architecture, the distance between sensors and computing units is greatly reduced, and by processing data at the sensing end, redundant data transmission is minimized, improving the overall performance of the sensing system. For agricultural scenarios requiring the deployment of large-scale low-power wireless sensor networks, edge computing sensors are expected to play an important role in time-sensitive applications. Therefore, developing deep learning models with relatively low computational requirements that can be used at sensor edge nodes is crucial.

#### 5.2.3 Artificial intelligence (AI)-driven advanced sensor development and intelligent application

First, applying artificial intelligence technology assists in the development of specific, highly sensitive sensing materials, significantly shortening the research and development cycle. Artificial intelligence and machine learning can serve as powerful auxiliary tools, predicting and screening the physical and chemical properties and key parameters of advanced sensing materials from large datasets, thus accelerating the discovery and validation efficiency of new materials, and providing new possibilities for the development of high-performance sensing materials. Secondly, applying artificial intelligence technology to construct intelligent systems that integrate multiple sensors. In actual agricultural production, it often requires the deployment of many heterogeneous sensors, resulting in messy data with poor inter-data flow. With the help of artificial intelligence technology, multi-level, multi-space information complementarity and optimized combination processing of various sensors can be achieved. Efficient screening, processing, and analysis of massive data, realizing visual data collection and precise analysis, is key to building future intelligent agricultural monitoring systems. Moreover, with the rapid advancement of smart agriculture, the integration of big data from ultra-sensitive sensors and artificial intelligence technologies such as machine learning will significantly drive the development of next-generation intelligent sensing systems (Zhu et al., 2020). Concurrently, as interactions between humans and machines increase, the fusion of Virtual Reality (VR) or Augmented Reality (AR) technologies with sensors is becoming a trend, resulting in comprehensive interactive systems within three-dimensional spaces. These interactive systems, enhanced by artificial intelligence sensors, will offer farmers more immersive scenario experiences.

### 5.3 Summary

As the primary means of obtaining crop planting data, crop sensors have infiltrated various application scenarios in intelligent agriculture. To meet the growing demand for data monitoring in intelligent agriculture, new sensing materials, manufacturing processes, and integrated components are continuously being developed, and the accuracy and reliability of sensors are constantly improving. This review systematically examines the major advancements in sensors for crop cultivation and their applications in intelligent agriculture. We summarize the key technologies of advanced sensors in smart farming: micro-nano sensing technology, flexible electronics, micro-electro-mechanical systems (MEMS) technology, hyperspectral sensing technology, and wireless sensor network technology. We also systematically summarize the application status and frontier progress of new advanced crop sensors like crop hormone sensors, crop nutrient sensors, crop moisture sensors, and crop growth sensors. We provide the material design, sensing mechanisms, and performance parameters of these sensors and discuss the pros and cons of each type. Meanwhile, we have introduced the latest research progress of multimodal sensors. This review provides key knowledge and a better understanding of the new development of advanced sensors for crop cultivation, revealing the challenges and trends of future smart agriculture research. It aims to inspire grasping the development trend of sensor technology in intelligent farming and selecting scientific and technological breakthrough points.
