# Supplementary material for: New horizons in smart plant sensors: key technologies, applications, and prospects
Source: Front Plant Sci. 2025 Jan 7;15:1490801. doi: 10.3389/fpls.2024.1490801 (PMC11747371; doi:10.3389/fpls.2024.1490801)
Supplement: Supplementary file 1 [file DataSheet1.docx]

Supplementary Information for

New Horizons in Smart Plant Sensors: Key Technologies, Applications, and Prospects

*Fucheng Zhang^1^,* *Denghua Li^1,2,3*^, Ganqiong Li^1,2,3*^, Shiwei Xu^1,2,3^*

^1^Agricultural Information Institute of Chinese Academy of Agricultural Sciences, Beijing 100081, P. R. China

^2^Key Laboratory of Agricultural Monitoring and Early Warning Technology, Ministry of Agriculture and Rural Affairs, Beijing 100081, P. R. China

^3^Research Center of Agricultural Monitoring and EarlyWarning Engineering Technology, Beijing 100081, P. R. China

***** Denghua Li, Email: [lidenghua@caas.cn](mailto:lidenghua@caas.cn) ; Ganqiong Li, Email: [liganqiong@caas.cn](mailto:liganqiong@caas.cn)


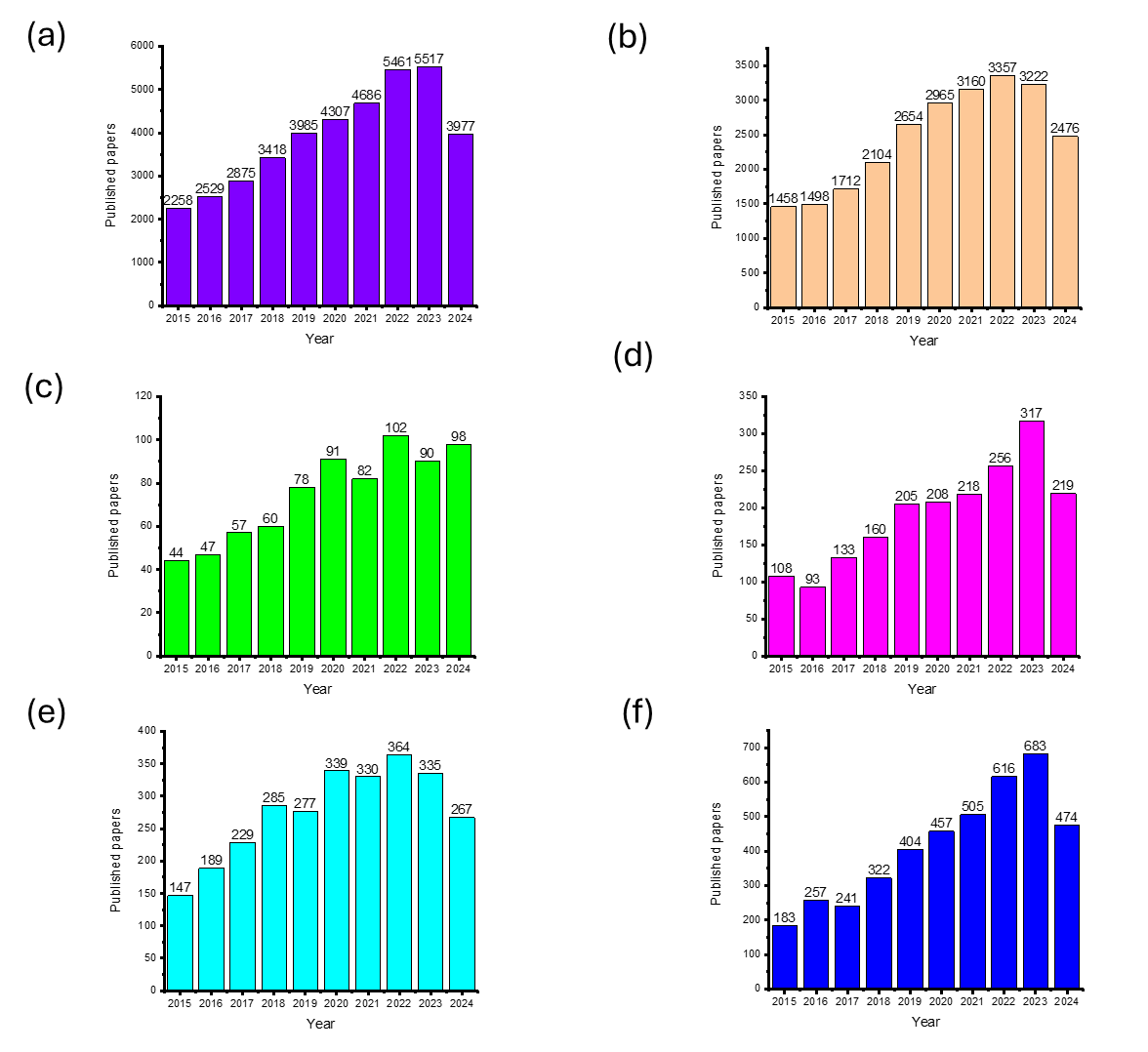


Figure.S1. Bibliometric analysis of different kinds of sensors published over the past decade using the Web of Science database. The deadline for statistics in 2024 is November 20, 2024. (a) Plant Sensors; (b) soil sensors; (c) Crop Hormone Sensors; (d) Crop Nutrient Sensors; (e) Crop Moisture Sensors; (f) Crop Disease Monitoring Sensors.
